# Targeting restoration sites to improve connectivity in a tiger conservation landscape in India

**DOI:** 10.7717/peerj.5587

**Published:** 2018-10-02

**Authors:** Trishna Dutta, Sandeep Sharma, Ruth DeFries

**Affiliations:** 1 Department of Ecology, Evolution, and Environmental Biology, Columbia University, New York, NY, USA; 2 Wildlife Sciences, Faculty of Forest Sciences and Forest Ecology, Georg-August Universität, Göttingen, Germany; 3 Smithsonian Conservation Biology Institute, Front Royal, VA, USA; 4 Workgroup on Endangered Species, J.F. Blumenbach Institute of Zoology and Anthropology, Georg-August-Universität, Göttingen, Germany

**Keywords:** *Panthera tigris*, Barrier, Connectivity, Restoration, Mitigation, Central India

## Abstract

**Background:**

Maintaining and restoring connectivity between source populations is essential for the long term viability of wide-ranging species, many of which occur in landscapes that are under pressure to meet increasing infrastructure needs. Identifying barriers in corridors can help inform conservation and infrastructure development agencies so that development objectives can be achieved without compromising conservation goals. Here, we use the tiger landscape in central India as a case study to identify barriers, associate them with existing infrastructure, and quantify the potential improvement by restoring or mitigating barriers. Additionally, we propose an approach to categorize linkages based on their current status within and between Protected Areas (PAs).

**Methods:**

We generated a hybrid landuse-landcover map of our study area by merging datasets. We used least-cost methods and circuit theory to map corridors and generate linkage metrics. We mapped barriers and used the improvement score (IS) metric to quantify potential improvement by restoring or mitigating them. Based on criteria that represent the status of corridors between-PAs and populations within-PAs, we ranked linkages into one of four categories: Cat1—linkages that currently have high quality and potential for tiger connectivity and should be maintained, Cat2W—linkages where focus on habitat and tiger populations may improve connectivity, Cat2B—linkages where focus on reducing barriers between PAs may improve connectivity, and Cat3—linkages where effort is needed to both reduce barriers between PAs and improve tiger populations and habitat within PAs. We associated barriers with infrastructure and present maps to show where restoration or mitigation measures can be targeted to have the highest potential impact.

**Results:**

We mapped 567 barriers within 30 linkages in this landscape, of which 265 barriers intersect with infrastructure (694 km of roads, 150 km of railway, 48 reservoirs, 10 mines) and 302 barriers are due to land-use or gaps in forest cover. Eighty-six barriers have both roads and railways. We identified 7 Cat1, 4 Cat2w, 9 Cat2b, and 10 Cat3 linkages. Eighty surface mines and thermal power plants are within 10 km of the least-cost paths, and more coal mines are closer to connectivity areas where linkages are narrow and rank poorly on both axes.

**Discussion:**

We present spatial and quantitative results that can help conservation practitioners target mitigation and restoration efforts. India is on the path to rapid economic growth, with infrastructure development planned in biodiversity-rich areas. The mitigation hierarchy of avoiding, minimizing, and offsetting impacts due to proposed development projects can be applied to corridors in this landscape. Cross-sectoral cooperation at early stages of project life-cycles to site, design, and implement solutions can maintain connectivity while meeting infrastructure needs in this rapidly changing landscape.

## Introduction

Wildlife conservation is increasingly focused at landscape scales, an approach that requires reconciliation of human needs with conservation priorities within and outside protected areas (PAs). Many PAs around the world are situated in human-dominated landscapes, where small sizes and increasing isolation between PAs limits the viability, persistence, and gene flow for many species (DeFries et al., 2005; Chundawat et al., 2016). For example, large mammalian carnivores have large area requirements, low densities, complex land-tenure systems, and long dispersal distances. These traits do not match the size and distribution of existing PAs, where most conservation effort is often directed (Chundawat et al., 2016). Long-distance movement is potentially impacted by barriers such as roads and surface mines outside PAs (Kerley et al., 2002; Cristescu, Stenhouse & Boyce, 2016).

Tigers (*Panthera tigris*) are a flagship conservation-dependent species that currently occur only in 7% of their historic range (Dinerstein et al., 2007). Presence of tigers is closely associated with tropical moist broadleaf forests and tropical dry forests in South-Asia and temperate broadleaf and mixed forests in the Russian Far-East (Sanderson et al., 2010). Tiger conservation landscapes (Wikramanayake et al., 1998; Dinerstein et al., 2007) consist of small and isolated PAs embedded in a mosaic of natural and anthropogenic land-use activities. Fifty-seven percent of the world’s tiger population is found in India, of which about 35% live outside PAs (Jhala, Qureshi & Gopal, 2015). India also supports 1.3 billion people and aspires to achieve an economic growth of 8% through urbanization, infrastructure upgradation, and energy extraction (Ministry of Finance, 2016). Several such development projects are proposed within potential tiger corridors (Dutta et al., 2015b; Habib et al., 2016), and the lack of a comprehensive national landuse policy (Department of Land Resources, 2013) risks unplanned, unmitigated, and permanent diversion of forestland.

Connectivity between PAs may be accomplished by a variety of approaches such as protection of corridors, restoration of degraded habitats, construction of mitigation structures, or land-purchase along corridors. Conservation practitioners employ two primary strategies to promote connectivity (i) focusing on conserving areas that facilitate movement (e.g., legally upgrading or protecting stepping-stone PAs along movement routes), and (ii) focusing on restoring connectivity across areas that impede movement (e.g., by removing barriers or building wildlife-friendly crossings) (McRae et al., 2012). In order to secure inviolate source populations, the government of India has been rapidly upgrading PAs to Tiger Reserves (PAs designated specifically for tiger conservation that receive increased funding and resources from the government). In just 8 years (2009–2016), eighteen PAs were upgraded to Tiger Reserves, and at-least eight others are under consideration (National Tiger Conservation Authority (NTCA), 2014). Although securing source areas and upgrading PAs is necessary in the face of increasing pressures on forestlands, PAs themselves are often too small and isolated to support long-term persistence of tigers (Chundawat et al., 2016). There has been considerably less effort at strategically identifying and removing barriers (areas that disrupt connectivity) along potential corridors. In many cases, avoiding new infrastructure through critical areas, removing or building mitigation structures across existing barriers, or restoring degraded habitat along corridors may provide an economically viable alternative to more traditional conservation actions such as land acquisition (McRae et al., 2012). Identifying barriers and quantifying potential impacts of mitigation and restoration measures can increase options for managers in determining where and how to invest scarce resources.

Here, we use the tiger conservation landscape in central India as a case study to identify barriers, categorize them by infrastructure type, and quantify the potential improvement by restoring or mitigating these barriers. Additionally, we propose an approach to target barrier-restoration by combining the improvement-score of individual barriers with the linkage category measured by the status of tiger populations within-PAs and the quality of connectivity between-PAs.

### Study area

Central India consists primarily of tropical dry deciduous (*Tectona grandis*) and tropical moist deciduous (*Shorea robusta*) forests (Champion & Seth, 1968) that harbor a rich assemblage of flora and fauna. The study landscape of 384,508 km^2^ is spread mainly across three states—Madhya Pradesh (MP), Maharashtra (Mh), and Chhattisgarh (Cg) (Dutta et al., 2015b), and contains 16 PAs (PA area ranges from 87 km^2^ to 3,188 km^2^). Central India is a global-priority landscape for tiger conservation (Dinerstein et al., 2007) and contains about 31% of India’s tiger population (Jhala, Qureshi & Gopal, 2015). The elevation ranges from 52 to 1,396 m and a majority of the forested area is along hilly tracts. Headwaters of the major peninsular rivers—Narmada, Tapti, and Ken originate in or around tiger-bearing forests in this landscape.

Agriculture, cattle rearing, and collection of forest products are important sources of livelihood for the people in this landscape. The region is also rich in coal and mineral deposits (Indian Bureau of Mines, 2017). Several infrastructure projects such as the construction and expansion of transportation networks, mining for coal and minerals, and building reservoirs for water security and hydropower are planned in the landscape (Habib et al., 2016; Ministry of Finance, 2016; Indian Bureau of Mines, 2017). Major transportation infrastructure that connects the far ends of the country passes through the tiger conservation landscape in central India.

## Methods

Using open-source data on landuse-landcover (LULC), population density, roads and railways, reservoirs, and surface mines (Table S1), we (1) generated a resistance surface for potential tiger movement, (2) mapped corridors and extracted linkage metrics, (3) mapped, quantified, and identified barriers, (4) identified infrastructure underlying each barrier, and (5) generated a ranking scheme to categorize linkages.

### Data sources

LULC—We generated a hybrid LULC layer for central India by merging relevant datasets after evaluating the accuracy of one national and four global LULC products (Bontemps et al., 2009; Hansen et al., 2013; Jun, Ban & Li, 2014; Tateishi et al., 2014; Roy et al., 2015) (details in S1). To this layer, we added two features that are barriers to tiger movement (a) reservoirs and dams (Lehner et al., 2011) and (b) surface mines and thermal power plants (TPP). We obtained mining areas from (Fernandes, 2012) and added 211 polygons digitized on Google Earth (26 TPPs and 185 surface mines, of which 79 are coal mines). Henceforth, we refer to mines and TPPs as mines. The final hybrid LULC map was at 30 m spatial resolution with eight classes: forest, degraded cover (that included scrub and degraded forest), barren land (abandoned shifting cultivation and barren land), open water, agriculture, human settlements, reservoirs, and surface mines.

Population density—We used the Landscan (2013) raster dataset (Bright et al., 2013), a global human population dataset at 1 km resolution. We calculated the population density and averaged the values using a 3 × 3 window. The resulting population density layer (mean 260, range 0–39,303) was categorized into five quantile-classes for further analysis.

Transportation infrastructure—We downloaded vector data on roads and railways from Open-Street Map (2015) and categorized the road network into highways (National or State), primary, and secondary roads. The resulting road layer had 4 categories (highways, primary roads, secondary roads, no roads) and rail layer had 2 categories (rail, no rail).

All vector data layers were rasterized and all layers resampled to 30 m resolution to generate the resistance surfaces using GNARLY utilities (McRae, Shirk & Platt, 2013). We then coarsened the resolution of the resistance layer by taking the average resistance in a 3 × 3 window so that the final cell size for connectivity analysis was 90 m. We coarsened the cell size to minimize any possible artifacts due to difference in formats and resolutions of the multiple underlying layers (Theobald, 2005).

### Creating the resistance surface

Resistance surfaces model the relationship between species movement and environment and are based on several assumptions about the ease of movement of animals through different landcovers (Spear et al., 2010). Due to the lack of large-scale movement, genetic, or occurrence data (Zeller, McGarigal & Whiteley, 2012) for tigers in the public-domain, we used previous resistance values based on expert opinion (Dutta et al., 2015b) as a starting point and developed 17 iterations of resistance surfaces to test sensitivity of models. We used four broad scenarios with three sub-scenarios each for LULC classes (total 12 scenarios) and five scenarios of layer weights following Rathore et al. (2012) (Fig. S1). We selected the resistance scenario that most closely resembled the consensus resistance surface from the LULC scenarios. Similarly, we selected the weighting scenario that most closely resembled the consensus raster for the layer weighting scenarios. Briefly, we compared the number of cells that were classified as corridor or non-corridor in the different scenarios using 300 random points and selected the a-priori scenario that had the highest correlation with the consensus raster. We also tested the impact of resistance and weighting scenarios on linkage mapping (details are presented in S2).

### Connectivity modelling

We used least-cost mapping and circuit theory modelling to delineate corridors, map least-cost paths (McRae & Kavanagh, 2011), and estimate linkage metrics (corridors and linkages are used synonymously in this study). Cost-weighted distance (CWD) surfaces were used to create least-cost corridors and least-cost paths (LCP) that connect patches (PAs in this case) in a landscape (Adriaensen et al., 2003). A least-cost corridor has a width that varies, whereas LCP is a one-pixel wide path between two patches that represents lowest inter-patch cost value. Effective resistance is a circuit-theory derived linkage metric that measures the relative isolation of the PAs while accounting for the availability of multiple movement routes (McRae et al., 2012). Details on how these metrics were used are presented later in the linkage categorization section.

### Mapping and characterizing barriers

(a) Mapping barriers: We used Barrier Mapper (McRae, 2012a; McRae et al., 2012) to identify, map, and quantify the potential improvement by restoration (e.g., reforestation) or mitigation (e.g., over-or-underpass across roads) of barriers. This tool measures the change in cumulative resistance before (LCD) and after (LCD’) restoring a certain area in an iterative moving window analysis. During each iteration, the resistance of the cells within a user-defined radius is set to 1, and cumulative resistance is measured. This allows for the calculation of a metric called improvement score (IS) represented as IS = LCD −LCD’.

The improvement score (IS) is interpreted as the connectivity improvement that would result from restoring the search radius. The radius of the analysis corresponds to the size of barriers one is interested in restoring. We used a variable search window radius to detect barriers at radii from 100 m to 2,000 m with 500 m increments to detect restoration possibilities at different scales and to test the sensitivity of our results. We present results from the 500 m radius here as it is representative of ongoing restoration efforts in the landscape (Anonymous, 2016) and summarize other results in S3. We divided the IS values into four classes using the quantile classification that represented four categories of barriers viz., moderate, medium, high, and very-high IS.

(b) Characterizing barriers: We used a series of overlay analysis and zonal statistics to characterize and associate barriers with underlying features. We first identified if a barrier intersected with any infrastructure or if it was only due to gaps in forest cover. If the barrier overlapped with some kind of infrastructure, we identified what kind of infrastructure (road, rail, mines, reservoirs) was present in the barrier.

(c) An illustration of barrier mapping: As an illustration of the applicability of this approach, we demonstrate the impact of restoring a small patch of 5*2cells (450 m long × 180 m wide) along a barrier in the Bor-Tadoba linkage. Bor is a small PA (<100 km^2^) with a breeding population of tigers surrounded by agriculture and development whereas Tadoba is larger PA (∼614 km^2^) with several coal-mines along its western periphery.

### Categorizing linkages

We ranked each linkage on two axes: (a) Between-PA status—measured by the resistance and width of intervening linkage, and (b) Within-PA status—measured by the tiger population in the PA-pair following (Doerr, Barrett & Doerr, 2011). We then plotted the between and within-PA ranks and categorized each linkage as belonging to one of four quadrants. Below is a detailed explanation of this process:

For between-PA status, we used (a) *CWD: LCP* ratio which measures the resistance along the path of least resistance. This ratio specifically indicates the average resistance encountered along the single most-optimal path between the PAs. This measure is derived from least-cost mapping, and lower ratios indicate higher quality (low resistance) linkages (WHCWG, 2010; McRae & Kavanagh, 2011; Dutta et al., 2015b); and (b) *CWD: effective Resistance (CWD: EffRes)* ratio measures the resistance along multiple routes within a corridor (Jones, Schindel & Scott, 2015). Effective resistance is estimated using circuit theory, measures total resistance between PA-pairs, and unlike the LCP which measures quality along one path, accounts for multiple or wide corridors. Low EffRes values indicate wide, low resistance linkages, therefore higher *CWD: EffRes ratios* indicate higher quality of the linkage. Higher ranks corresponded to higher quality linkages, i.e., linkages with lower CWD:LCP had higher ranks, whereas those with higher CWD:EffRes ratios had higher ranks.

When combined, these metrics represent the overall quality of linkages. Linkages that have low resistance along the least-cost path and have multiple low resistance routes are more likely to have a higher potential of facilitating tiger movement between the PAs. We derived the overall between-PA status by averaging the rank on these two metrics.

For within-PA status, we calculated a simple gravity model derived index previously used in connectivity analysis (Forman & Godron, 1986; Linehan, Gross & Finn, 1995; Lee & Oh, 2012). It is calculated as

\begin{eqnarray*}{G}_{ab}=({N}_{a}\ast {N}_{b})/{D}_{ab}^{2} \end{eqnarray*}

where *G*_*ab*_ is the potential interaction between PAs_*a*_ and _*b*,_ *N*_*a*_ and *N*_*b*_ are the population sizes within the two PAs, and *D*_*ab*_ is the Euclidian inter-PA distance. We derived population sizes from the available density estimates and PA-area (Ramesh et al., 2013; Jhala, Qureshi & Gopal, 2015) and assigned a low density of 0.05 tigers per 100 sqkm for two PAs (Phen and Noradehi) which did not have published population estimates.

G_*ab*_ is based on the notion that close-by areas with large tiger populations have higher potential of animal movement and therefore more connectivity. It measures the interaction between the PAs as a function of the population size within the PAs, while accounting for the distance between the PAs. Retaining the distance is important in this case, because although two PAs may have large populations, if they are far from each other, the functional connectivity between them may be highly unlikely.

Combining ranks: We plotted the rank for each linkage along the two axes of between-PA and within-PA status. Linkages that ranked high on both dimensions (Category1) could be considered as high-quality linkages that currently have the highest potential of animal movement and therefore need to be maintained. Category3 linkages rank poorly in both dimensions, and therefore need several multi-pronged interventions to make them more effective for tiger movement. Category2 linkages that rank on only one-dimension need to be preferentially improved along the low-ranked dimension (Category 2W needs more within-PA efforts and Category2B needs more between-PA efforts).

Finally, we converted the barrier IS raster to polygons, aggregated adjacent barrier polygons, and populated each polygon with the maximum IS in that barrier. We used this polygon layer to link individual barriers of different IS values with the linkage categories.

### Validating results

We did not have access to empirical data on tiger movement to validate our results. However, we used alternative analytical methods to evaluate if disparate methods based on different underlying principles led us to similar results. To validate the barriers, we mapped pinch-points, which are areas of high current flow through a narrow area, indicating bottlenecks or the lack of alternative pathways (McRae et al., 2008; McRae, 2012b). A spatial overlap of barriers and pinch-points would support our barrier-mapping exercise. To compare the categorization of linkages, we created a Minimum Spanning Tree (MST), a frequently applied approach to identify the minimum set of linkages to protect (Urban & Keitt, 2001). Linkages that are ranked highly on the categorization plot would also be expected to be connected in the MST. We expect these results to be refined and validated with more field data in the future.

## Results

**Figure 1:**
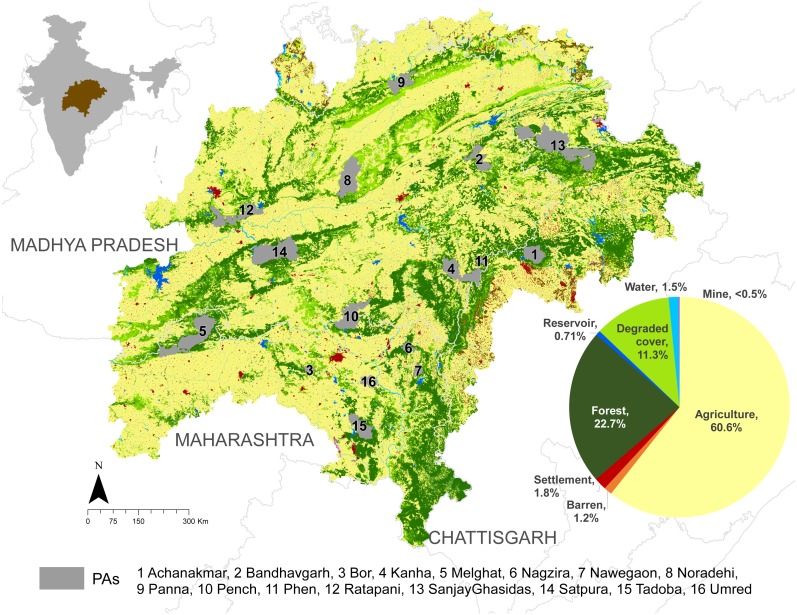
Map of the study landscape showing the landuse-landcover patterns, state boundaries, and protected areas. A majority of the landscape is dominated by agriculture. Details are provided in S1.

The LULC map (Fig. 1) generated through the hybrid approach resulted in a landscape dominated by agriculture (61%), forest (23%), and degraded cover (11%).

For the 12 LULC resistance iterations, 47% of cells in the landscape were never classified as a corridor. Of the cells that were classified as corridors, ∼52% were identified as corridors in more than 9 resistance schemes and ∼46% were consistently identified in all 12 schemes. Results across the five weighting scenarios were also consistent, ∼81% of cells were consistently identified as corridors. The consensus raster was most similar to the resistance scenario where forest is least resistant to tiger movement (Pearson’s *r* = 0.96) and the weighting scheme where population and LULC have twice the weight of transportation (Pearson’s *r* = 1) (S2).

The connectivity analysis resulted in thirty-one linkages, of which thirty linkages consistently appeared in the 17 sensitivity test runs (S2). We dropped the linkage (Bor-Satpura) unique to this result and used the remaining 30 linkages for further analysis. On average, Euclidian distance (EucDist) between the 16 PAs is 102.96 km (SD 51.95), and CWD is 1,331.45 km (SD 950.52). The longest LCPs are between Panna-SanjayGhasidas (∼547 km, EucDist ∼166 km) and Panna-Noradehi (∼500 km, EucDist ∼112 km). The shortest LCPs are between Kanha-Phen (∼16 km, EucDist ∼7 km) and Nagzira-Nawegaon (∼48 km, EucDist ∼18 km). Linkage quality (as indicated by CWD:LCP) is poorest for Satpura-Ratapani (42.8), Bor-Umred (23.5), and Bor-Tadoba (16.8) and highest for Kanha-Phen, Achanakmar-Phen (both ∼1), and Kanha-Pench (1.6). The CWD:EffRes ratios are high for Melghat-Satpura (177.3) and Kanha-Pench (161.8) indicating wider, low-resistance linkages, and low for Nawegaon-Nagzira (∼46), Achanakmar-Bandhavgarh (54.5) and Achanakmar-Sanjay (55.2) indicating narrow, high-resistance linkages. The gravity index is highest for Kanha-Pench indicating highest potential interaction and lowest for Bandhavgarh-Phen and Achanakmar-Phen indicating very low potential interaction.

**Figure 2:**
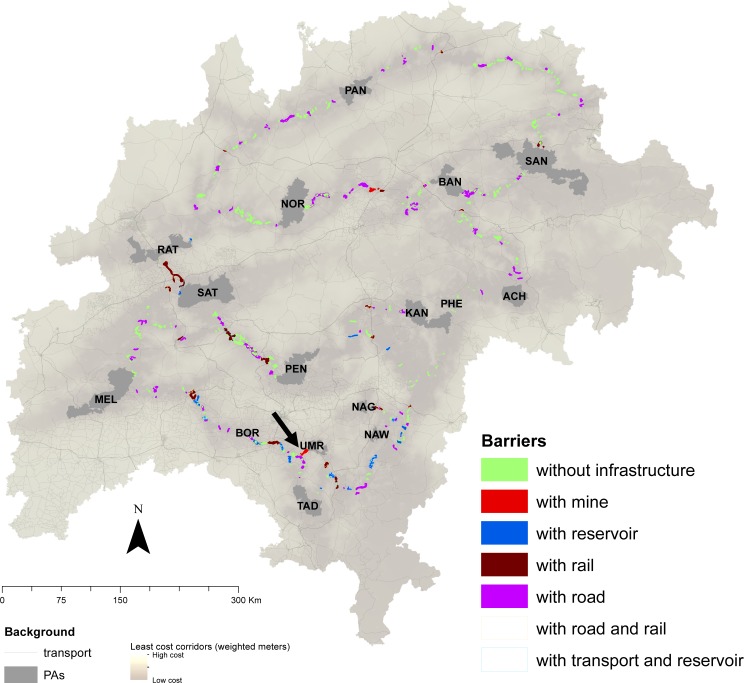
Barriers classified according to the feature they intersect. There are a total of 567 barriers, of which 265 barriers have infrastructure where mitigation and restoration measures may be employed, and 302 barriers that are due to land-use or forest breaks, where restoration projects can be targeted. One barrier had all four infrastructure features (indicated by the arrow), 5 barriers had reservoirs, roads, and rail, and several barriers had two features (roads and rail in 86, reservoirs and roads in 25, and reservoirs and rail in 7 barriers). PA abbreviations: ACH (Achanakmar), BAN (Bandhavgarh), BOR (Bor), KAN (Kanha), MEL (Melghat), NAG (Nagzira), NAW (Nawegaon), NOR (Noradehi), PAN (Panna), PEN (Pench), PHE (Phen), RAT (Ratapani), SAN (SanjayGhasidas), SAT (Satpura), TAD (Tadoba), UMR (Umred).

We mapped a total of 567 barrier polygons in this landscape (Fig. 2) which contained agriculture (1,260 km^2^), forest (1,821 km^2^), degraded cover (1,183 km^2^), open-water (150 km^2^), barren-land (17 km^2^), and settlements (15 km^2^). 265 barriers are due to infrastructure (180 barriers have ∼694 km roads, 47 barriers have ∼150 km rail, 35 barriers have ∼48 reservoirs, three barriers have ∼10 mines), whereas 302 barriers are due to forest-cover gaps. One barrier had all four infrastructure features, five barriers had reservoirs, roads, and rail, and several barriers had two features (roads and rail in 86, reservoirs and roads in 25, and reservoirs and rail in seven barriers). Barriers mapped at smaller search radii were closer to the least-cost paths and were a subset of barriers mapped at larger radii (S3).

**Figure 3:**
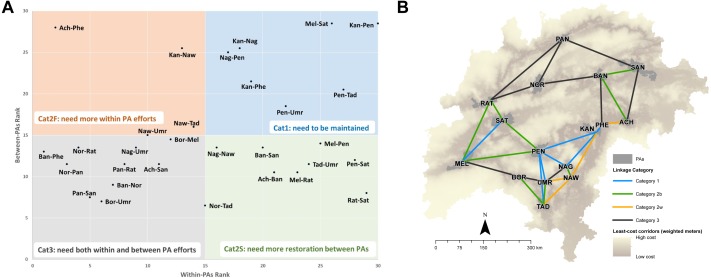
Categorization of linkages in central India. The plot in (A) shows the relative ranking of a linkage on the two axes of between-PA and within-PA status. Category1 linkages (blue) rank high on both axes, Category3 linkages (grey) rank poorly on both axes, while Category2 linkages (orange and green) rank high on only one axis. The map in (B) shows the location of the different linkages in the landscape. Abbreviations in the categorization plot: Ach(Achanakmar), Ban(Bandhavgarh), Bor(Bor), Kan(Kanha), Mel(Melghat), Nag(Nagzira), Naw(Nawegaon), Nor(Noradehi), Pan(Panna), Pen(Pench), Phe(Phen), Rat(Ratapani), San(SanjayGhasidas), Sat(Satpura), Tad(Tadoba), Umr(Umred).

**Figure 4:**
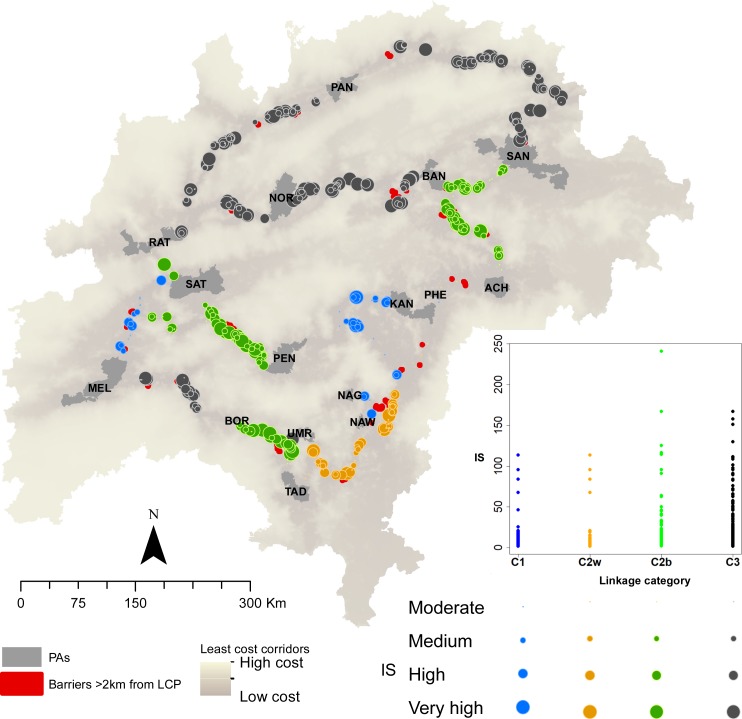
The location and improvement scores of barriers in different linkage categories. The size of circles indicate the improvement scores (IS), and the colors indicate the category. Barriers away from LCPs are shown in red. The number and IS of barriers in the different categories of linkages are presented in the plot, which shows a large number and high-IS barriers in Category3 and Category2B linkages.

In the categorization plot (Fig. 3) there are seven Category1 linkages, four Category2W linkages (low rank in within-PA status), nine Category2B linkages (low rank in between-PA status), and 10 Category3 linkages. Most linkages from Kanha rank highly on both axes, whereas most linkages from Panna, Noradehi, and Bor rank poorly on both axes (Fig. 3B). Numerically, most barriers are in Category3 linkages (no. barriers = 310), several barriers in Category2B linkages (no. barriers = 160) and Category1 linkages (no. barriers = 99) and few barriers in Category2W linkages (no. barriers = 53) (Fig. 4). Further, most high IS barriers are in Category3 and Category2B linkages (86 and 37 barriers have IS in the top 20% of IS).

**Figure 5:**
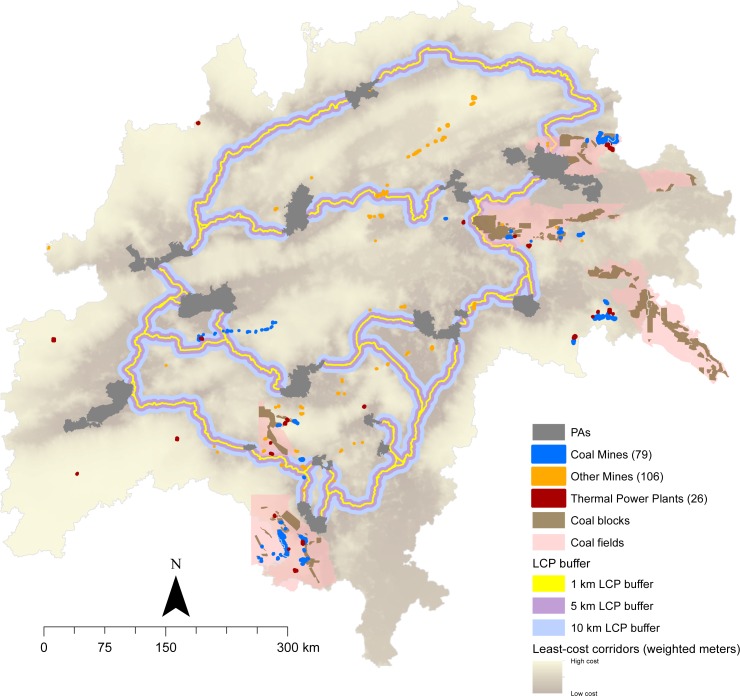
The location of existing thermal power plants and mines and potential mining areas represented by coal blocks and fields in relation to the least-cost paths in the landscape. By carefully selecting restoration sites and avoiding pinch points, mining sector can fund and contribute to restoration efforts that will maintain an interconnected landscape while meeting human needs.

Fourteen, 51, and 80 mines (surface mines and TPPs) are within a distance of 1, 5 and 10 km of the LCP respectively (Fig. 5). More mines are closer to connectivity areas in the southern and north-eastern part of the landscape, where linkages are narrow and rank poorly on both categorization axes.

**Figure 6:**
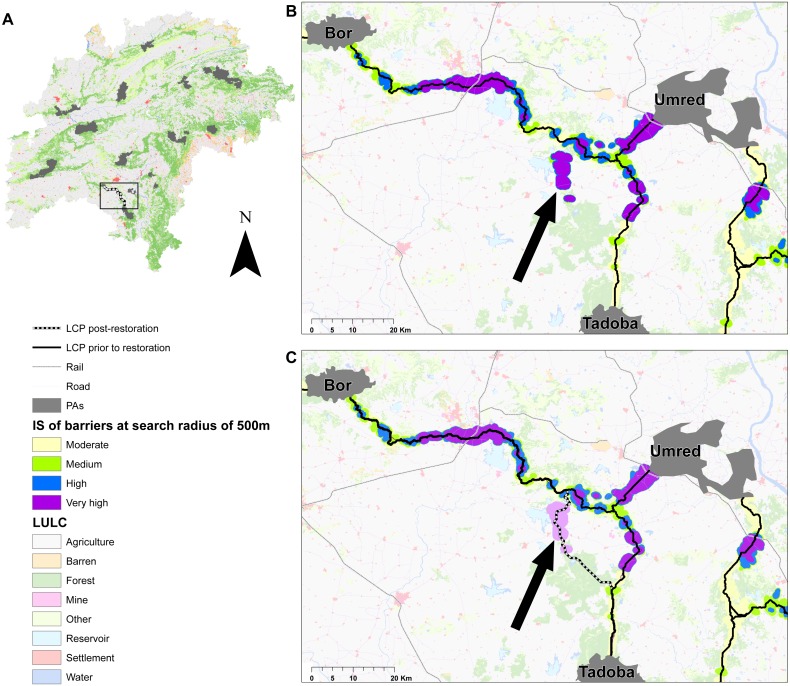
Illustration of the barrier simulation application in the Bor-Tadoba linkage. Illustration of the barrier mapping application. (A) shows the inset of the landscape. (B) shows a close up of the Bor-Tadoba (Category2b) linkage where a strip of 180 × 450 m was restored in the area indicated by the arrow. (C) shows that post-restoration (shown in light purple), an alternative LCP was identified along which a second corridor could be maintained. Similar approaches may be applied by simulating restoration in barriers away from the LCP in other linkages in order to increase redundancy in connectivity.

There were several barriers detected away from existing LCPs (Fig. 4) and restoring one such barrier in the Bor-Tadoba linkage resulted in an alternate LCP being created, thus providing redundancy in this Category2B linkage (Fig. 6). Restoration efforts in barriers away from existing LCPs could provide opportunities to add redundancy and conserve connectivity in this region (S3).

Alternative approaches corroborate key results- pinch points and barriers are spatially coincident, and a majority (12 out of 15) linkages connected in the MST belonged to Category1 and Category2 (S4).

## Discussion

Conserving landscape connectivity is a vital component of conservation (Beier & Noss, 1998; Bennett, 1999; Crooks & Sanjayan, 2006; Hilty, Jr & Merenlender, 2012) that supports biodiversity persistence (Olds et al., 2012) and ecosystem resilience (Heller & Zavaleta, 2009). Connectivity maps often represent vision and goals for the future (McRae et al., 2012) which may be accomplished by a variety of approaches such as protecting corridors, restoring degraded habitats, building mitigation structures, or buying land along corridors. Barrier detection helps increase alternatives in the conservation toolbox that can be applied by managers to identify where and how to invest scarce resources to target-areas that have high potential impact.

We present spatial and quantitative results that can help conservation practitioners focus mitigation and restoration efforts within the landscape at different scales. The improvement scores of individual barriers are quantitative measures to guide restoration and mitigation efforts at a small scale (e.g., for single or few linkages). When barrier IS is combined with linkage category, it can guide landscape-level decisions on restoration and mitigation. Category1 linkages presently have the highest potential of supporting animal movement, and efforts should be towards protecting and strengthening these linkages by allocating resources to maintain and augment existing linkages and prevent loss in connectivity due to new infrastructure. In Category2B linkages, restoration and removal of barriers in the corridor would significantly improve connectivity, while habitat and population management within linked PAs in Category2W linkages would enhance interaction and potential connectivity between the PAs. A combination of restoration and barrier-removal in corridors, and within-PA enhancements are required to improve connectivity in Category3 linkages. The categorization of linkages presented here are not meant to ignore Category3 linkages, but rather inform pragmatic decision-making about restoration efforts.

Previous studies have established functional connectivity in this landscape for several large carnivores (Dutta et al., 2013; Dutta et al., 2015a; Sharma et al., 2013a; Sharma et al., 2013b; Yumnam et al., 2014). Cross-sectoral conservation groups working in this landscape concur on the need to develop a landscape-level conservation approach that integrates social, economic, and ecological needs in the region (DeFries, Sharma & Dutta, 2016). By identifying specific infrastructure features within the barriers, we provide clear opportunities for conservation managers to work with the multiple responsible agencies such as the Ministry of Mines and National Highway Authority of India.

A barrier may be associated with natural (e.g., natural lakes), or anthropogenic features (e.g., transportation networks, mines, man-made reservoirs). Restoration of forest cover where it is discontinuous, such as sections in Bandhavgarh-Sanjay, Pench-Satpura can help improve the permeability of the landscape. Barriers due to transportation networks such as along sections of Bandhavgarh-Noradehi, Bor-Melghat may be mitigated by planning strategically designed over-and-underpasses (Glista, DeVault & DeWoody, 2009). Most barriers are due to a combination of landscape features (Fig. 2). For example, in several parts of Kanha-Pench, Nagzira-Nawegaon corridor where roads and railways lines are adjacent; road and railways intersect a forest gap in Bor-Tadoba and Pench-Satpura corridor; roads, railways, and marble mines are present along a forest-gap in the Bandhavgarh-Noradehi corridor. Because most linkages have multiple barriers, removing one high impact barrier may result in the emergence of an alternate path and provide opportunities to add redundancy in connectivity. Several barriers were detected away from the current LCPs, especially at larger search radii (S3) and restoration along a small section in the Bor-Tadoba linkage (Fig. 6) illustrates that restoration efforts could lead to the creation of alternative low-cost paths, thus adding redundancy and improving connectivity in this landscape.

Surface mining is a prominent development activity across the world which often impacts biodiversity-rich areas. Energy needs are expected to increase across the world, and coal is projected to be India’s most important energy source in the future (India Planning Commission, 2013). Coal mining alone accounted for 65% of the total forest land diverted for mining from 2007–2011 (Centre for Science and Environment (CSE), 2012). Central India has rich deposits of coal and 80 surface mines and TPPs are within 10 km of LCPs (Fig. 5). Open-cast mining for coal and other resources is particularly prevalent in Pench-Satpura, Bandhavgarh-Noradehi, Pench-Bor-Umred-Tadoba, and Achanakmar-Bandhavgarh-SanjayGhasidas corridors (Fig. 5). There is a trend towards underground mining in forest areas, but several coal blocks and fields intersect the corridors, where future mining expansion, intensification, and associated transportation infrastructure and settlements may be anticipated (Fig. 5). Mining permissions could be avoided in areas with barriers and pinchpoints (S4), and government-mandated compensatory afforestation can be directed to barrier-restoration (Fig. 3).

Eighty-six barriers had both kinds of transportation networks (road and rail). Transportation infrastructure to meet the needs of people and energy is likely to increase globally (Laurance et al., 2014), which is a proven barrier to movement and gene-flow for many terrestrial carnivores across the world (e.g. Sawaya, Kalinowski & Clevenger, 2014). At least 11,000 km of roads and railways are planned for construction through other tiger landscapes (World Wildlife Fund (WWF International) & Dalberg, 2016) e.g., in the Terai-Arc (NH125) and Kaziranga (NH37) landscape (Srivastava & Tyagi, 2016). This also presents an opportunity to engage the transportation sector and fund well-designed mitigation measures in conservation landscapes, which have been shown to be effective for other carnivores (Sawaya, Kalinowski & Clevenger, 2014). Globally, new transportation infrastructure is increasingly planned to avoid high-quality habitats or designed to minimize and mitigate adverse effects (Van der Ree, Smith & Grilo, 2015). Incorporating mitigation plans before construction is more cost effective than retrofitting existing highways and railways (Glista, DeVault & DeWoody, 2009; Weller, 2015). Transportation infrastructure is necessary to meet India’s development goals, but developers and conservationists could collaborate and experiment with combinations of engineering (e.g., building crossing structures,) and behavioral (e.g., lower traffic speeds, traffic convoys, or higher toll rates across barriers) solutions to reduce and mitigate impacts.

The analysis presented here is specific for tigers but maintaining connectivity for this umbrella species can help conserve and restore connectivity for other species (Breckheimer et al., 2014). The barrier-mapping and categorization of linkages presented in this study are based on static data, but the underlying data are in reality dynamic. As tiger populations change and new infrastructure is built, the categorization, barrier location and IS can also be expected to change. Future development of user-friendly decision making tools can help facilitate the adoption of such research by managers and planners. We present barriers detected at 500m search radius, but it is important to note that more barriers are detected as the search radius is increased. We do not expect these results to be a blue-print for restoration or mitigation initiatives in the region, but rather to initiate cross-sectoral engagement for wildlife friendly infrastructure development India. We anticipate that finer-scale studies will need to be conducted on a site-by-site basis and only then can real on-the-ground solutions be provided. Further, it is important to note that these results are a snap-shot in time, whereas both tiger populations and the status of linkages are dynamic processes.

Ultimately, any decision-making to support connectivity depends on a strong legal and policy framework that allows for species and habitat protection outside the PA network. Although no specific law defines, protects, or prohibits development within or around wildlife corridors in India, several policies have provisions that allow states to legally recognize important habitats as eco-sensitive zones, conservation reserves, community reserves, or biodiversity heritage sites (Srivastava & Tyagi, 2016). Mechanisms to fund barrier restoration exist through Corporate Social Responsibility (CSR) funds for the private sector (Anonymous, 2016) and government (CAMPA—Compensatory Afforestation Fund Management and Planning Authority).

The issues presented in this study are applicable to many species and landscapes across India and the world. There are several ambitious efforts and plans to connect PAs at a continental scale (e.g., the Yellowstone to Yukon Conservation Initiative in North America and Mesoamercian Biological Corridor (Shadie & Moore, 2007)), and national scale (e.g., Bhutan and Australia (Wildlife Conservation Division, 2010; Commonwealth of Australia, 2012)). Along similar lines, India has developed guidelines to mitigate the impact of linear infrastructure on wildlife (WII, 2016). However, the implementation of connectivity conservation on the ground has been slow and challenging. This failure to translate connectivity research into conservation action has been termed as the ‘research-implementation’ gap, and several broad challenges and solutions to bridge this gap have been outlined in Keeley et al. (2018). By generating visual maps that combine barrier-mapping with linkage categorization, our study can contribute towards three of these identified solutions: (i) to build partnerships across public, private and individuals, (ii) to develop a common vision of connected landscapes that integrates social, ecological, and economic outcomes, and (iii) to base implementation on sound science that use empirical data on animal movement to validate, plan, and prioritize connectivity zones.

The development vs. conservation debate is not unique to India or tigers, and multi-pronged options to improve connectivity between source populations are necessary in most biodiversity-rich areas of the world. It is estimated that 22 trillion USD will be invested to support increased infrastructure development by 2030, mostly in developing countries (International Energy Agency (IEA), 2007). An often employed approach to meet development and biodiversity needs is the mitigation hierarchy, which is a decision-making framework designed to address impacts on biodiversity and ecosystem services through (1) seeking to avoid impacts wherever possible, (2) minimizing or restoring impacts, and (3) by offsetting any unavoidable impacts (Phalan et al., 2017). Kiesecker et al. (2010) recommended a shift from traditional project-by project mitigation of impacts that underestimates the cumulative impacts of multiple concurrent development projects in an area to a landscape level which offers more flexibility in applying the mitigation hierarchy.

Our study can help bridge the conservation-development divide by offering spatially-explicit quantitative results on potential improvements by targeting restoration and mitigation measures across a variety of infrastructure types and are applicable at a landscape scale. Corridor and barrier maps could be used to evaluate environmental clearance for projects that are within or close to corridors or barriers, and also to redirect afforestation funds and efforts to barriers.

## Conclusion

Our study is first of its kind in tiger-range countries to spatially identify and quantify barriers in a priority conservation landscape, and can aid landscape-level conservation in the region. We identified barriers, quantified the potential improvement by restoration or mitigation measures, associated barriers with underlying landscape features, and ranked the linkages into different categories. Site-specific finer-scale analysis will have to be undertaken when implementing mitigation measures, but our results present an overview of opportunities to improve connectivity conservation in the region. We aspire to help build partnerships by providing quantitative and spatial information on barrier mitigation and restoration possibilities. The maps we produced could help in initiating and building cross-sectoral cooperation such as between the scientific community, forest department, and agencies responsible for the different infrastructure (highway, railway, water, and mining). We are optimistic that this study could help towards building a holistic vision for a connected landscape while integrating conservation and economic development. We urge developers, engineers, and wildlife experts to collaborate and engage at early stages to design and implement solutions that maintain connectivity for wildlife while accommodating development needs for infrastructure. Similar approaches may be useful for other species in rapidly changing landscapes across the world.

##  Supplemental Information

10.7717/peerj.5587/supp-1 Supplemental Information 1 Supplementary material

## Additional Information and Declarations

### Data Availability

The following information was supplied regarding data availability: All data used in this study along with their sources are listed in Table S1 and are freely available.
