# Supplementary material for: Targeting restoration sites to improve connectivity in a tiger conservation landscape in India
Source: PeerJ. 2018 Oct 2;6:e5587. doi: 10.7717/peerj.5587 (PMC6173158; doi:10.7717/peerj.5587)
Supplement: Supplemental Information 1 [file peerj-06-5587-s001.docx]

**SUPPLEMENTARY MATERIAL**

**TARGETING RESTORATION SITES TO IMPROVE CONNECTIVITY IN A TIGER CONSERVATION LANDSCAPE IN INDIA**

**SUPPLEMENTARY S1**

**Generation of hybrid LULC layer for central India**

During our initial assessments we found that readily available LULC layers were not very accurate. Therefore, we merged available datasets to create a more accurate LULC layer for this landscape. We used six broad categories of land-cover types – agriculture, forest, barren, degraded cover, open water, and settlement for our analysis. We use these cover types for resistance mapping in subsequent analyses.

**Method:** We assessed and combined different LULC datasets presented in TableS1. We calculated and compared the overall accuracy and error rates (omission and commission) for our designated land cover classes. We used 470 randomly generated ground truth points across the region and visually identified the cover type on google earth imagery between 2014-2016. To be conservative in the resistance mapping, we wanted to be conservative and avoid false positives for habitat with low resistance (forest) and false negatives for habitats with high resistance (settlement and agriculture). We set the following criteria for each different LULC category to derive a hybrid land-cover map.

Agriculture and settlement: Select the land cover data set with highest commission error and lowest omission error (avoid false negatives).

Forest, degraded cover, water: Select the land cover data set with highest omission error and lowest commission error (avoid false positives).

If the omission or commission errors were similar, we selected the data with higher overall accuracy.

**Result:** Overall accuracy for our study region was highest for the global land cover dataset developed by China (Jun et al 2014) and the India specific vegetation map by Roy et al (2015), so we derived data for individual land cover classes from these data (Table S1). Class-wise error rates for these two datasets are presented in Table S2. We used Globeland dataset as a base map, selected settlements and barren classes from Roy et al (2015). In addition, we used Hansen et al (2013) to select cells with forest cover greater than 33 % (calculated mean forest cover in forest ground truth points). The accuracy and error rates for the resulting hybrid LULC map are presented in TableS3. In addition to these classes, we added two features that are relevant to tiger movement - dams from the GRAND database (Doll et al 2003), and 211 surface mines and thermal power plants that we digitized on google earth. The final hybrid map is at 30m spatial resolution with 8 classes (Fig 1 in the main manuscript).

Table S1: Details and sources of datasets used in this analysis. The overall accuracy is also stated for LULC data we validated for Central India.

|  | Name of the data | Producing agency | Year | Resolution | Details | Reference | Overall accuracy for CI |
| --- | --- | --- | --- | --- | --- | --- | --- |
| LULC | Global Land Cover by National Mapping Organizations  (GLCNMO) | Geospatial Information Authority of Japan, Chiba University and collaborating organizations | 2008 | 500m | Global dataset, 20 land cover classes | http://www.iscgm.org/gm/glcnmo.html | 42% |
| LULC | Globeland 30 | National Geomatics Center, China | 2010 | 30m | Global dataset, 10 classes | Jun et al (2014) Nature 514. http://www.globallandcover.com/GLC30Download/index.aspx | 61% |
| LULC | Vegetation type map of India | Indian Space Research Organization, India | 2010 | 24m | Vegetation cover map for India, a total of 100 classes | Roy et al (2015) New  vegetation type map of India prepared using satellite remote  sensing: comparison with global vegetation maps and utilities.  Int J Appl Earth Obs Geoinf 39:142–159. doi:10.1016/j.jag.2015.  03.003 | 54% |
| LULC | GlobCover | European Space Agency | 2009 | 300m | Global dataset, 22 land cover classes | Bontemps S., Van Bogaert E., Defourny P., Kalogirou V. and Arino O., “GlobCover 2009 – Products Description Manual”, version 1.0, December 2010. December 2010. (http://ionia1.esrin.esa.int/). | 49% |
| LULC | Global Forest Change 2000–2014 | University of Maryland | 2014 | 30m | Percent forest cover per pixel | Hansen et al (2013) High-Resolution Global Maps of 21st-Century Forest Cover Change. Science 342:850-853.  http://earthenginepartners.appspot.com/science-2013-global-forest/download_v1.2.html | NA |
| Dams | GRanD | Global Water System Project | 2003 | Vector | This database compiles reservoirs with a storage capacity of more than 0.1 km³ | Döll et al (2003). A global hydrological model for deriving water availability indicators: model tuning and validation. Journal of Hydrology 270: 105–134.  http://www.gwsp.org/products/grand-database.html | NA |
| Roads and railways |  | Open Street Map (2015) |  | Vector | User generated map of roads and railways | https://www.openstreetmap.org | NA |
| Population |  | Oak Ridge National Laboratory (ORNL) | 2013 | 1 km | Global Population Database | LandScan (2013) High resolution global population data set copyrighted by UT-Battelle, LLC, operator of Oak Ridge National Laboratory under Contract No. DE-AC05-00OR22725 with the United States Department of Energy. | NA |

Table S2: Error rates in the contributing datasets along with their error rates. Final selection for the particular LULC category are highlighted in bold.

| Class | Omission | Commission | Overall accuracy | Globeland  -------------  Vegetation Type map of India |
| --- | --- | --- | --- | --- |
| Agriculture | 0.15 | 0.33 | 67.31% | **Globeland** |
|  | 0.64 | 0.31 | 69.41% | Vegetation Type map of India |
| Forest | 0.22 | 0.44 | 55.71% | **Globeland** |
|  | 0.12 | 0.58 | 41.51% | Vegetation Type map of India |
| Degraded cover | 0.72 | 0.78 | 21.82% | **Globeland** |
|  | 0.79 | 0.83 | 17.31% | Vegetation Type map of India |
| Barren | 0.89 | 0.84 | 16.13% | Globeland |
|  | 0.76 | 0.80 | 20.00% | Vegetation Type map of India |
| Open water | 0.47 | 0.06 | 94.44% | **Globeland** |
|  | 0.12 | 0.19 | 80.95% | Roy |
| Settlement | 0.46 | 0.27 | 73.08% | Globeland |
|  | 0.24 | 0.21 | 79.10% | Vegetation Type map of India |

Table S3: Error rates and overall accuracy in the final hybrid LULC layer

| **Class** | **Omission** | **Commission** | **Overall accuracy** |
| --- | --- | --- | --- |
| Agriculture | 0.38 | 0.26 | 73.91% |
| Forest | 0.24 | 0.39 | 61.29% |
| Degraded cover | 0.67 | 0.72 | 28.00% |
| Barren | 0.69 | 0.83 | 17.07% |
| Water | 0.47 | 0.06 | 94.44% |
| Settlement | 0.09 | 0.24 | 76.19% |

**SUPPLEMENTARY S2**

**Generating the consensus resistance surface**

First we tested a total of 17 scenarios of resistance and weighting schemes - 12 scenarios to test the effect of varying resistance values to different land cover types, and 5 scenarios to test the weighting of different layers while preparing the resistance maps. A summary of the resistance and weighting scenarios is presented in TableS4. Within each resistance scenario, we had three sets of resistance values. We generated values for corridor/non-corridor values for 300 random points to assess similarity between the scenario and consensus raster (pixels where 10 or more runs delineated as corridor). Despite differences in the outputs across different runs, overall there is a general agreement in the corridor delineation across variations in resistance and weighting scenario. FigS1 and FigS2 represent the summary of the resistance and weighting scenarios respectively.

TableS4: Summary of the four broad scenarios to test the effect of varying resistance values to different land cover types, and 5 scenarios to weight the different layers while preparing the resistance maps (17 total variations of resistance surface). Resistance scenario 1b and weighting scenario 2 (in bold) were the most similar to the consensus raster and used for analysis in this study.

| Scenarios | Resistance | Layer weighting | |
| --- | --- | --- | --- |
| 1 | **Forest is least resistant** | All three layers have same weight | Pop + LULC + Transport |
| 2 | Degraded cover has lowest resistance | **Population and LULC have twice the weight of transportation** | 2*(Pop + LULC) + Transport |
| 3 | Agriculture is not very resistant | Pop has twice the weight of LULC and transport | 2*Pop + LULC + Transport |
| 4 | Agriculture is very resistant | LULC ha twice the weight of LULC and transportation | Pop + 2*LULC + Transport |
| 5 |  | Transportation has twice the weight of LULC and Pop | Pop + LULC + 2*Transport |

**Impact of varying resistance values for LULC on corridor delineation**

We set up the sensitivity test for resistance to LULC types under four broad scenarios. Within each scenario, we tested three different combinations of resistance values.

(1) forest is least resistant to tiger movement and every other land cover has a higher resistance,

(2) forest and degraded cover (scrub and degraded forest) are equally suitable for animal movement outside of PAs,

(3) agriculture offers more resistance to animal movement than forest, but is not unsuitable to movement, and

(4) agriculture is highly resistant to tiger movement.

**Method:** We used Gnarly utilities (McRae et al. 2013) to create the resistance surfaces and Linkage Mapper(McRae & Kavanagh 2011) to generate cost-weighted surfaces and delineate corridors in the landscape.

In order to test the impact of resistance, we did the following:

(i) used a cut-off of value of 200000 cost-weighted distance to delineate the landscape into corridor or non-corridor (ii) calculated the number of times a pixel was classified as a corridor or non-corridor pixel across the different test runs (iii) created a raster that consisted of cells which were classified as corridor 10 or more times across the 12 runs (FigS1) for the resistance. Then, in order to select which resistance scenario was most similar to the consensus raster, we then generated 300 random points and extracted corridor/non-corridor attributes to these points. We compared the different scenarios and selected the resistance scheme that was most similar to the consensus raster. We followed the same procedure to select the weighting scheme.

**Result:** Despite differences in the outputs across different runs, overall there is a general agreement in the corridor delineation across variations in resistance scenario.

When comparing between the different resistance scenarios, Scenario 2 which included forest as well as degraded and scrub forest to have least resistance resulted in the maximal area marked as corridors (~149,511 sqkm), followed by Sc3 (129,319 sqkm) where agriculture had low resistance, Sc1 (112,168 sqkm) wherein forest cover was the least resistant to tiger movement, and finally Sc4 (94,799 sqkm) where agriculture had high resistance. Using the random points, we found that Sc1b (forest has the least resistance, Scheme b) was the most similar to the consensus raster (pixels where 10 or more runs delineated as corridor). Therefore, we selected this resistance scenario for our final analysis.

Figure S1: Summary of the resistance scenarios. The table shows the three resistance value schemes under each of the four broad resistance scenarios. Mapped corridors are shown for each of the 12 scenarios on the left and the large map on the right shows the consensus resistance raster and the random points used to generate the correlation of each test run with the consensus raster.


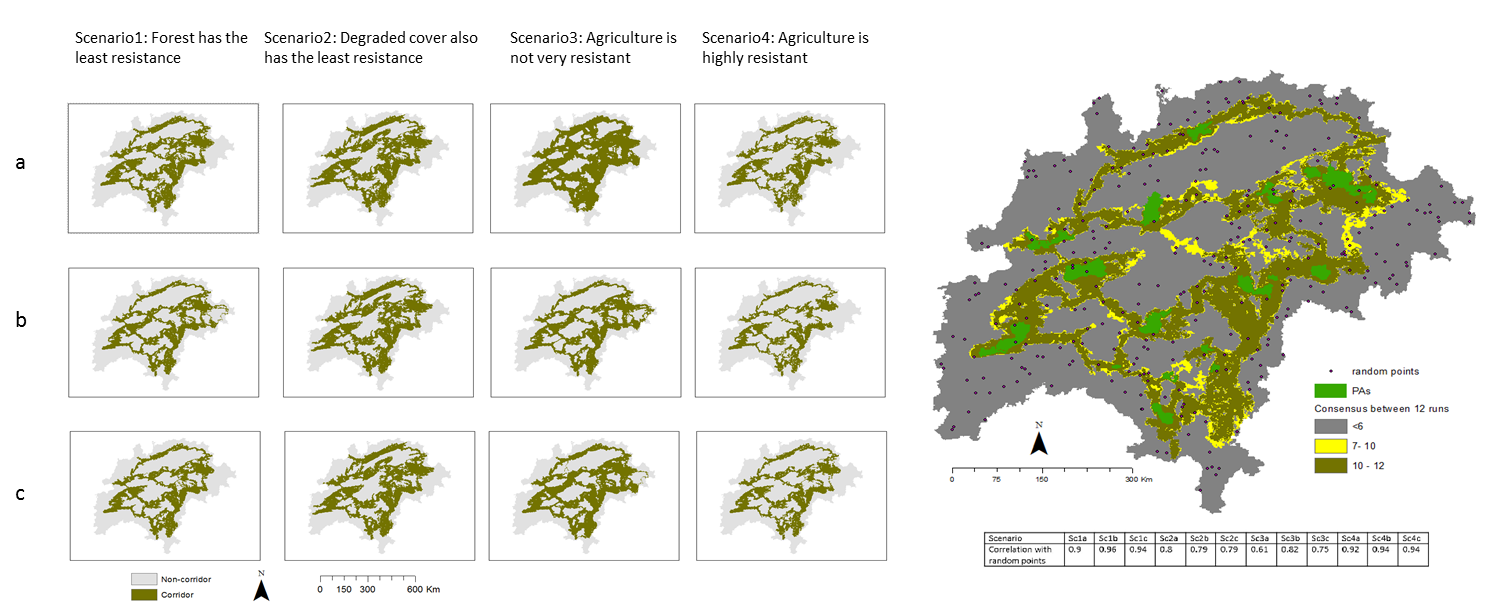


**Impact of varying weightage of different layers on corridor delineation**

We weighted the three layers -LULC, transportation network (roadways and railways), and human population density in 5 scenarios:

(1) When all layers had the same weight,

(2) human population density and LULC had twice the weight of transportation networks,

(3) human population density was twice the weight of LULC and transport,

(4) LULC had twice the weight of human population density and transportation networks, and

(5) Transportation networks had twice the weight of LULC and human population density

**Method:** We used LULC, transportation network (roadways and railways), and human population density for our analysis. We used Gnarly utilities (REF) to create the resistance surfaces and Linkage Mapper (REF) to generate cost-weighted surfaces and delineate corridors in the landscape.

In order to test the impact of weighting schemes, we used the same approach as in the resistance sensitivity test. Briefly, we (i) used a cut-off of value of 200000 cost-weighted distance to delineate the landscape into corridor or non-corridor (ii) calculated the number of times a pixel was classified as a corridor or non-corridor pixel across the different test runs (iii) created a raster that consisted of cells which were classified as corridor in each of the 5 runs (FigS2) (iv) in order to select which resistance scenario was most similar to the consensus raster, we used 300 random points and extracted corridor/non-corridor attributes to these points. (v) We compared the different weighting scenarios and selected the one that was most similar to the consensus raster.

**Results:** Despite differences in the outputs across different runs, overall there is a general agreement in the corridor delineation across variations in weighting schemes. The weighting scheme where population density and LULC had twice the weight of transportation network was most identical to the consensus raster. Therefore, we selected this weighting scheme for our final analysis.


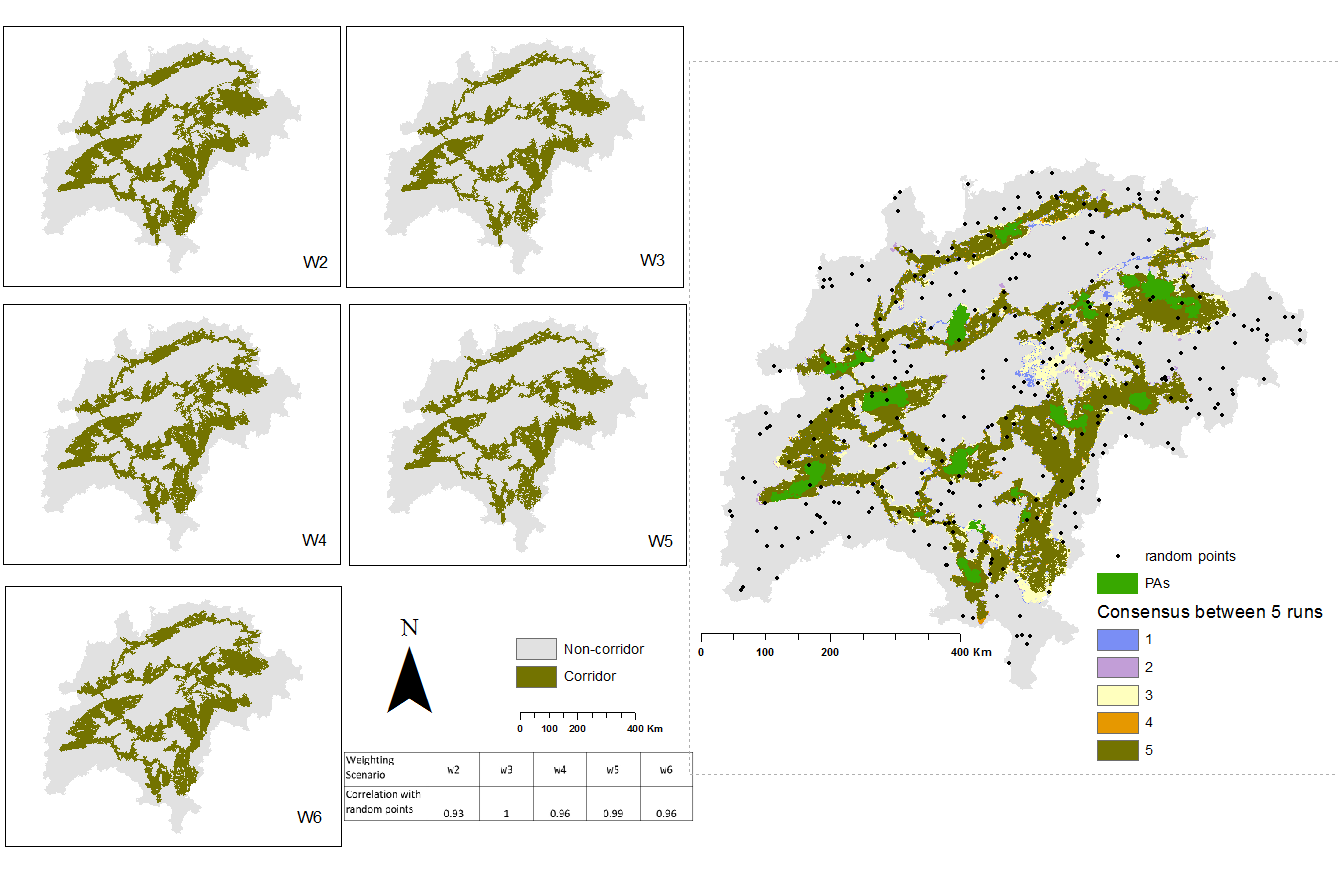


Figure S2: Summary of the weighting scenarios. The table shows the five weighting scenarios, mapped corridors are shown for each of the 5 scenarios on the left and the large map on the right shows the consensus resistance raster and the random points used to generate the correlation of each test run with the consensus raster.

**Impact of resistance and weighting scenarios on linkage mapping**

We used results from linkage mapping to identify which links were consistent across runs. We used these results to fine-scale our final analysis.

**Result:** A total of 39 possible linkages emerged during the sensitivity tests with different resistances and weighting scenarios. Thirty-two linkages and twenty-eight linkages appeared every time in the weighting tests and resistance tests respectively. Twenty-seven linkages appeared consistently in both weighting and resistance scenarios. FigS3 shows the linkages consistent across runs along with the linkages in the final run. In our final analysis, there was one linkage between Satpura and Bor (indicated by the orange arrow) that was not present in any of the sensitivity tests. We therefore discarded this linkage in our analysis. Several linkages that appeared only occasionally in the test runs were not present in the final run.


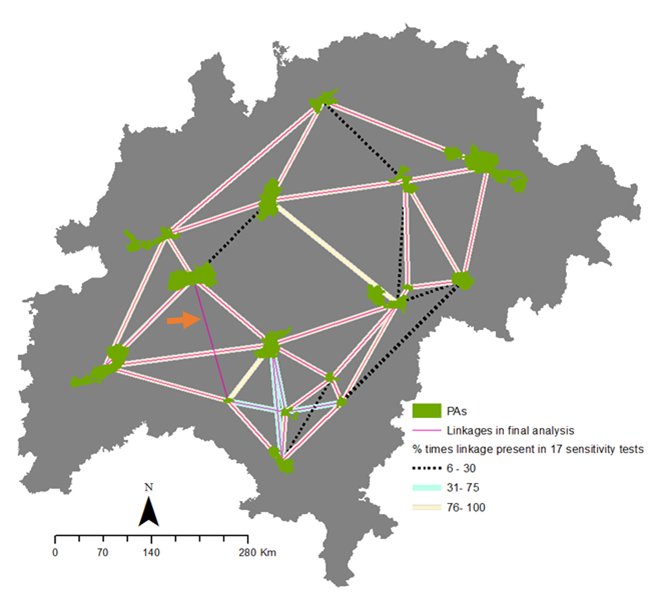


FigureS3: Linkages in the 17 different sensitivity runs. We conducted analysis with 30 linkages that appeared consistently in the test runs.

**SUPPLEMENTARY S3**

**Impact of varying search radii on barrier detection**

By modifying the search radius, one can detect barriers of different sizes. We used 5 different search window sizes- 100m, 500m, 1000m, 1500m, and 2000m.

**Methods:** We used the resistance maps generated for the linkage mapping exercise in the tool Barrier Mapper (McRae 2012a). This tool identifies the improvement score (IS) as the difference between the cumulative resistance along the optimal path before and after a user-defined area is restored. It is interpreted as the improvement in connectivity per unit area restored.

**Results:** Detected barriers across the various search radii were very similar. At smaller radii, most barriers were along or close to the least-cost path. As search radii increased, additional barriers further away from the least-cost path were identified.

Figure S4: Results of barrier detection with five search radii. Barriers detected were consistent, with more restoration opportunities detected at larger search radius.


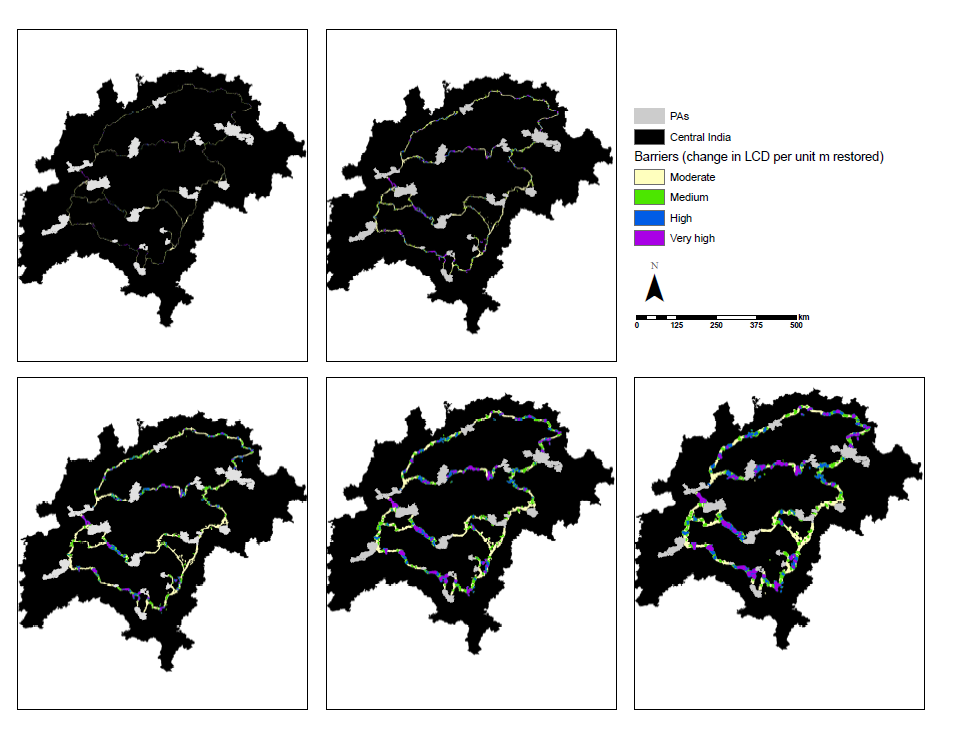


2000m

100m

500m

1000m

1500m

**SUPPLEMENTARY S4**

**Validation of barrier mapping and categorization of linkages**

Due to the lack of empirical data on tiger genetics or movement data, we used alternative analytical methods to compare the consistency of our methods.

**Methods:** We mapped pinch-points, which is measured as the current flow density per cell in the program Pinch-point mapper (McRae 2012b). Areas of high current density are sections where current flow is restricted to a very narrow area, suggesting the lack of alternative pathways. Pinch-points are therefore considered as bottlenecks to animal movement and any further loss in these sections would lead to disproportionate connectivity losses. A spatial overlap of barriers and pinch-points would support our barrier-mapping exercise.

To compare the categorization of linkages, we created a Minimum Spanning Tree (MST), a frequently applied approach to identify the minimum set of linkages to protect(Urban & Keitt 2001). Linkages that are ranked high on the categorization plot would also be expected to be connected in the MST. We expect these results to be refined and validated with more field data in the future.

**Results:** Mapped pinch-points and barriers were coincident (Fig S5) and nodes connected on the MST were also ranked highly (Category1 or 2) in the linkage categorization plot (FigS6). We expect the ongoing research in the landscape will further validate and improve these results.

*
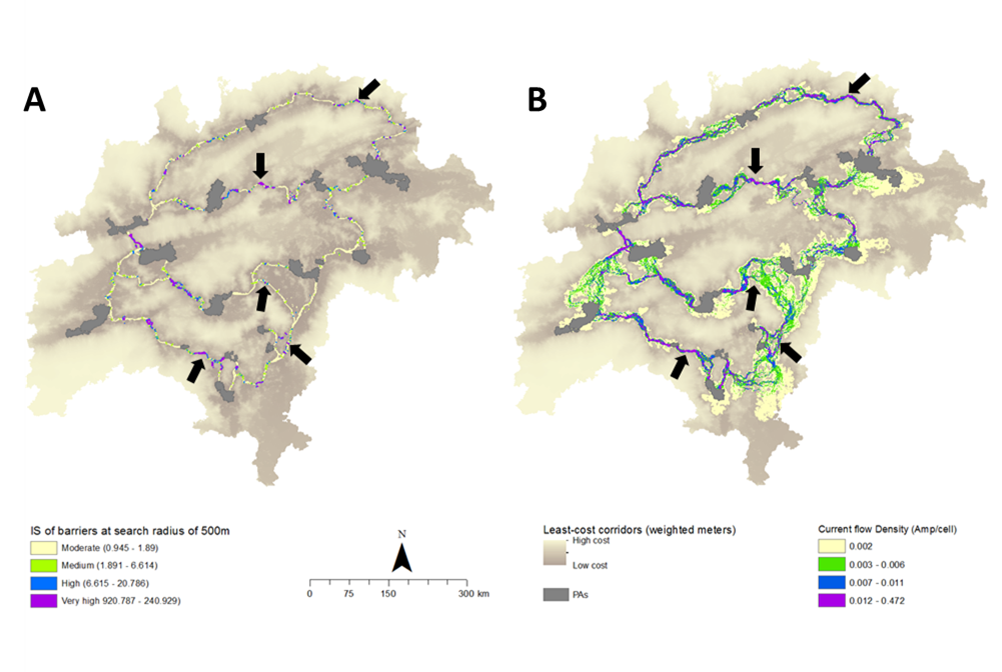
*

Figure S5: Comparison of mapped barriers (A) with pinch points (B). Many barriers are coincident with pinch-points, some of them are highlighted by the arrows.

Fig 3: Panel of barriers (3a) and pinch points (3b) in the landscape. We classified the output rasters for barrier IS and pinchpoints by quantiles. Arrows highlight some areas where high IS barriers overlap with pinchpoints where connectivity is already constricted. Barriers may present restoration and mitigation opportunities that can improve connectivity in the landscape.


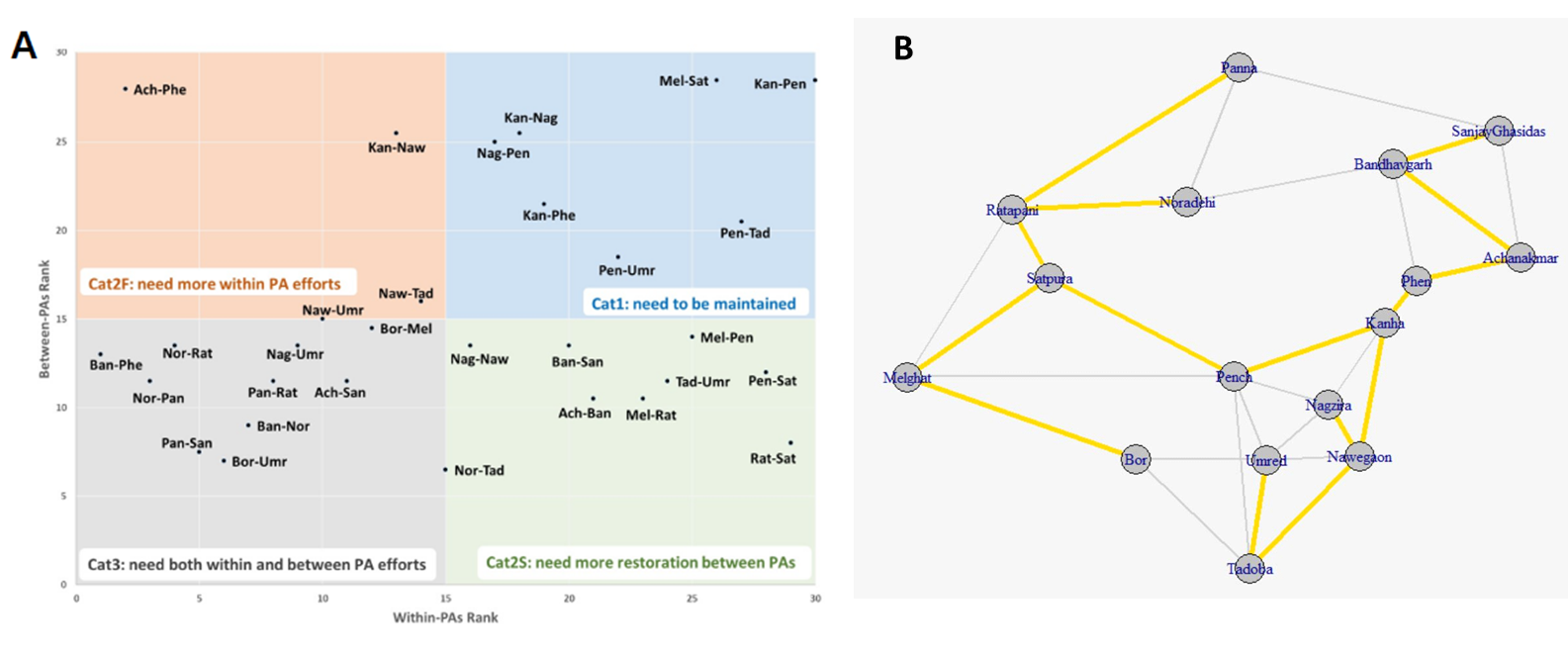
Figure S6: The categorization plot (A) shows the different linkage categories and the MST (B) shows the minimum set of linkages that need to be conserved. A majority (13 out of 15 MST linkages) Cat1 or 2 linkages (highlighted in bold in the categorization plot).
